# Risk of Thrombosis, Pregnancy Morbidity or Death in Antiphospholipid Syndrome

**DOI:** 10.3389/fcvm.2022.852777

**Published:** 2022-03-01

**Authors:** Martin Killian, Thijs E. van Mens

**Affiliations:** ^1^CIRI – Centre International de Recherche en Infectiologie, Team GIMAP, Université de Lyon, Université Jean Monnet, Université Claude Bernard Lyon 1, Inserm, U1111, CNRS, UMR530, Saint-Étienne, France; ^2^Internal Medicine Department, Saint-Etienne University Hospital, Saint-Étienne, France; ^3^Amsterdam UMC, Department of Vascular Medicine, Amsterdam Cardiovascular Sciences, Amsterdam Reproduction and Development, University of Amsterdam, Amsterdam, Netherlands

**Keywords:** pregnancy morbidity, obstetric antiphospholipid, antiphospholipid syndrome, venous thromboembolism (VTE), antiphospholipid antibodies

## Abstract

The antiphospholipid syndrome is an autoimmune disease characterized by thrombosis and pregnancy morbidity. The manifestations are caused by antibodies targeting cell membrane phospholipids and/or associated proteins. The triggers leading to these antibodies' production are unknown but recent work suggests cross-reactivity between the autoantigens and peptides produced by the intestinal microbiome. Work on how the autoantibodies could cause clinical manifestations implicates different mechanisms. Binding to surface proteins of different cell types can induce intracellular signaling leading to cell activation and tissue factor expression. Complement activation and neutrophil extracellular-traps are also involved, and recent evidence implicates endothelial protein C receptor-lysobisphosphatidic acid complex. Pregnancy is a high-risk situation for antiphospholipid syndrome patients due to the increased risk of thrombosis and obstetric complications. Epidemiological and clinical research on APS is hampered by heterogeneity in populations, testing and treatment strategies. About one in 10 to one in fifty APS pregnancies is complicated by thrombosis, despite treatment. Pregnant patients with prior thrombosis are prescribed therapeutic dose heparins and low dose aspirin. Without prior thrombosis a prophylactic dose is used. The most frequent obstetrical manifestation is recurrent early pregnancy loss. The association of APS antibodies with late pregnancy loss is stronger, however. Prevention of recurrence is achieved with aspirin and prophylactic dose heparin, although the evidence is of low certainty. The third obstetrical classifying manifestation comprises preterm delivery due to placenta-mediated complications and is treated in subsequent pregnancies with aspirin with or without prophylactic dose heparin, again based on low quality evidence. New therapies are under investigation.

## Introduction

Antiphospholipid syndrome (APS) is a rare autoimmune disease, whose key features are recurrent vascular thrombosis and obstetrical complications, but can also be responsible for thrombocytopenia, haemolytic anemia, cardiac valvular disease, renal thrombotic microangiopathy, neurological symptoms, cognitive impairment or pulmonary hypertension (1). It is also frequently associated with systemic lupus erythematosus, and its approximate prevalence is 40 per 100 000 individuals (2, 3).

APS-specific autoimmune response is targeting components of the cell membrane i.e., phospholipids (e.g., cardiolipin) and/or their associated proteins (mainly β2-glycoprotein-I [β2GPI]) in its phospholipid-bound “activated” open conformation which is exposing cryptic epitopes in its first domain (4–6). Antiphospholipid antibodies (aPL; see Table 1), have historically been described in 1983, in Syphilis, as well as in multiple infectious diseases since (20). In such infectious setting, aPL are usually thought of as transient and non-thrombogenic, however thombotic complications have been reported in a small number of aPL-positive infection cases, possibly in autoimmunity-prone individuals (21, 22). Interestingly, aPL have recently been reported in a significant proportion (up to 30–50%) of acute COVID patients, especially in severe cases, but it is still debated whether they could be contributing to the disease prothrombotic state independently of the several potentially confounding factors (23). Of note, the aPL epitope specificity is different in COVID (i.e., rarely targeting β2GPI domain I) (24), and the autoantibody persistence over time (≥ 2 positive testing, 12 weeks apart) seems to be absent in most COVID cases (23, 25), in line with what has been described in infection-related cases (26).

**Table 1:** Spectrum of the main autoantibodies associated with antiphospholipid syndrome.

**Specificity**	**LAC**	**aCL**	**Anti-β2GPI**	**Anti–β2GPI Domain 1**	**Anti-β2GPI/HLA-DR**	**Anti-PS**	**Anti-PT**	**Anti-PS-PT complex**	**Anti-Annexin V**	**Anti-Annexin II**
Known isotypes	NA	IgG, IgA, IgM	IgG, IgA, IgM	IgG	IgG	IgG, IgA, IgM	IgG, IgA, IgM	IgG, IgA, IgM	IgG, IgM	IgG, IgM
Approximate prevalence in definite APS	30–80% (7, 8)	10–50% (8, 9)	5–45% (9, 10)	50–60% (8, 11)	80% (12, 13)	45–85% (11, 14)	15–55% (15, 16)	25–75% (7, 8)	15–40% (7, 17)	25–40% (18, 19)
In Classification	Yes	Yes (IgG, IgM)	Yes (IgG, IgM)	No	No	No	No	No	No	No

*LAC, lupus anticoagulant; aCL, anti-cardiolipin; anti-PS, anti-phosphatidylserine; anti-PT, anti-prothrombin; PE, anti-phosphatidylethanolamine; NA, not applicable*.

Detecting aPL is primordial for diagnosing APS, but determining if these autoantibodies are culprits (aPL positivity with an APS-compatible clinical setting) or innocent bystanders (aPL positivity alone) can be complicated (23, 27). Classification criteria have been formulated during International Congresses on APS in Sapporo and Sydney, and subsequently published as consensus statements in 1999 (28) and 2006 (29), respectively. The 2006 revised classification criteria for definite APS are met when at least one clinical criterion (vascular thrombosis or pregnancy morbidity), and one biological criterion (Lupus Anticoagulant [LAC], IgM/IgG anti-cardiolipin [aCL], and/or IgM/IgG anti-2GPI positivity) are present. These criteria, which were never intended for diagnostic use, have significant drawbacks: non-inclusion of the less frequent but well-identified APS manifestations (30) or non-inclusion of “non-criteria” autoantibodies (e.g., anti-prothrombin, anti-annexin V, anti-phosphatidylserine…) (31, 32).

The objective of this mini review article is to provide a clear but concise summary regarding pregnancy-related complications in APS, particularly focusing on recent insights, research gaps and future concepts in the pathogenesis, epidemiology, prevention, and treatment of thrombotic and non-thrombotic manifestations.

## Origin of APS Autoantibodies

APS pathogenesis is thought to rely upon both genetic and environmental factors, which would explain why several microorganisms can trigger transient aPL, whereas only few predisposed individuals will develop definite APS (33). Like other autoimmune diseases, the exact trigger for autoantibodies is unknown. Several theories exist however, including recent work identifying an intestinal microbe as a source of cross-reactive antigens thought to trigger APS autoimmunity (34, 35). A comparison of known APS epitopes within β2GPI with intestinal microbiome metagenomic data, identified *Roseburia Intestinalis* as a gut microbe with “mimotope” peptides for both B and T-cells, and cross-reactivity was experimentally confirmed in humans and mice. Moreover, a *Roseburia Intestinalis*-induced APS phenotype was reported in APS-prone mice.

On another note, some non-β2GPI-specific aPL could be natural antibodies (i.e., polyreactive, non-immunization induced and B1 cell-secreted) (36), whose pathogenicity could be secondarily induced or enhanced by antigen-driven mutation (37).

Regarding the genetic background, different human leukocyte antigen (HLA) gene polymorphisms have been associated with the occurrence of certain types of aPL: HLA-DR5 and HLA-DRw53 with aCL and LAC; HLA-DPB1^*^0301 and HLA-DPB1^*^1901 with anti-β2GPI; HLA-DQB1^*^0301, HLA-DQA1^*^03, and HLA-DRB1^*^04 with anti-prothrombin; HLA-DRB1^*^08 with anti-annexin V, and HLA-DQB1^*^0301 with anti-phosphatidylserine (38). These findings suggest that the way these autoantigens—or microbial antigens, through molecular mimicry (21, 34)—are presented to the immune system, is important for the generation of the corresponding autoantibodies. Interestingly, another potential autoantibody-generating mechanism has been described for HLA class II molecules and their ability to aberrantly present cellular misfolded proteins [i.e., exposing cryptic epitopes (5) or creating neoantigens (12)] to the cell surface without processing to peptide (39). In line with this, anti-β2GPI/HLA-DR complex antibodies were recently reported in 83% cases of APS (12), and 20% cases of unexplained recurrent pregnancy loss (13).

## Pathophysiology of Thrombotic Manifestations

According to the 2006 revised classification criteria for APS, the “vascular thrombosis” criterion is met with the occurrence of ≥1 episode(s) of objectively (i.e., *via* appropriate imaging or histopathology) confirmed arterial, venous, or small vessel thrombosis, in any tissue or organ, excluding superficial venous thrombosis (29).

The exact underlying pathogenic mechanisms behind APS have not yet been fully elucidated (40), but multiple leads linking coagulation and autoimmunity have been described:

- aPL direct interference with the endogenous anticoagulant systems e.g., decrease in protein C/S and thrombin plasma levels (41).
- inhibition of β2GPI-stimulated fibrinolysis by anti-β2GPI autoantibodies (42).
- anti-β2GPI antibody-dependent activation of the classical complement pathway in the “standard” thrombotic manifestations of APS (43, 44), but also of the alternative pathways in its catastrophic form due to additional germline mutations in complement regulatory genes (45).
- autoantibody-mediated activation (including C5a and C5b9-related mechanisms) of endothelial cells (46– 48), platelets (48–52) and monocytes (53, 54), particularly leading to tissue factor pathway-dependent procoagulant activity *via* various [and sometimes paradoxical (55)] mechanisms (56).
- release of neutrophil extracellular traps (NETs) by activated neutrophils (57).
- endothelial protein C receptor (EPCR)-lysobisphosphatidic acid (LBPA) engagement by aPL, leading to thrombosis and driving dendritic cell interferon-α production for the expansion of aPL-secreting B1 cells (56).

These autoantibodies' pathogenic effects are frequently referred to as the “first hit,” inducing a persistent thrombophilic state, which requires a “second hit,” usually an inflammatory and/or a prothrombotic condition, to elicit the clinical manifestations (40). Pregnancy can be viewed as such, because of its well-described associated hypercoagulable state, including overlapping mechanisms such as acquired activated protein C resistance or increased tissue factor expression and activation (58).

## Pathophysiology of Pregnancy Manifestations

According to the 2006 revised classification criteria for APS, the “pregnancy morbidity” criterion is met with the occurrence of at least one of these events (without any alternative cause): (1) ≥1 unexplained death(s) of a morphologically normal fetus (≥10th week of gestation). (2) ≥1 premature births of a morphologically normal neonate (<34th week of gestation) because of eclampsia, severe pre-eclampsia or placental insufficiency. (3) ≥3 unexplained consecutive spontaneous abortions (<10th week of gestation) (29).

Interestingly, whereas high titres and multiple aPL positivity are usually associated with thrombotic manifestations in APS, low titres aPL have been frequently reported in obstetric APS (59, 60). The fact that high levels of β2GPI can be found in the placenta is a possible explanation for this, moreover direct effects (notably through complement, Toll Like Receptors and inflammasome pathways) on trophoblast cell and endometrium differentiation have been reported for aPL (61–65). The recently described anti-β2GPI/HLA-DR antibodies may have a pathogenic role in obstetric APS by inducing complement-dependent cytotoxicity-mediated damaging in vascular endothelial cells of the placental decidua (12). Similarly, the EPCR/LBPA complex is involved in aPL signaling in embryonic trophoblast cells, and using an anti-EPCR/LBPA-blocking antibody was protective from fetal loss in a relevant mouse model (56). Other non-criteria aPL have been reported in obstetric APS, including anti-Annexin antibodies (66) or aPL of the IgA isotype (67).

## Clinical Implications of Pregnancy in APS

A current or planned pregnancy demands careful counseling and therapeutic decision making in APS patients. Unfortunately, clinical research on APS is hampered by equivocal data from both epidemiological studies and clinical trials. General reasons for this include heterogeneity in APS testing, cut-off values, patient selection, and treatment protocols. The mainstay of treatment for pregnant APS patients—despite the evidence for a coexisting role of non-thrombotic processes in the pathogenesis—is anticoagulant therapy. This applies to both thrombotic and obstetric APS. Bleeding complications are the main drawback. Bleeding risk was investigated in a *post-hoc* analysis of one retrospective and one prospective cohort of pregnant APS patients receiving low dose aspirin (LDA) and/or low molecular weight heparin (LMWH) (68). The incidence of bleeding events was 25% in the retrospective cohort, with major bleedings, all early post-partum, occurring in 3% of pregnancies. In the prospective cohort only a single bleeding event (1.2%) was recorded. Major bleeding was defined as requiring intervention for hemostasis or blood transfusion, or during the peripartum period >500 mL. A control group was not included in this study, but the rates do not clearly exceed those in untreated pregnant women. Heparin-induced thrombocytopenia and allergic reactions also seem rare (69).

## Pregnancy-Related Venous Thrombosis

### Epidemiology

Pregnancy is a prothrombotic state, due to physiological changes in anatomy and circulating hormones and coagulation proteases (70). Hence, pregnancy forms an additional risk factor for thrombosis in APS patients. An estimated one in four thrombotic events in APS are pregnancy related (71). The absolute risk for thrombosis during pregnancy and the postpartum period is variously reported from 1 to 12% (72–74). Not all patients in these studies had APS according to the currently accepted criteria. The reported thrombotic events, mostly venous thrombosis, occurred under different treatment regimens including with and without heparins. Despite these limitations, pregnancy carries a high risk for thrombosis in APS. The risk is further determined by the patients' antibody profile. A high-risk profile comprises persistent positivity for LAC or a combination of at least two of the three aPL, with the general concept of higher titers conferring a higher risk (75). Another major risk factor is a previous thrombosis, and traditional venous thromboembolism (VTE) risk factors likewise apply to the pregnant APS patient.

Patients with purely obstetric APS also have an increased risk for future thrombotic events. Patients with recurrent miscarriage had a thrombotic event rate of 19.3% after a mean follow-up of 7.3 years in one study, with no thrombosis in the group with idiopathic recurrent miscarriage (76). Another case control study in obstetric APS patients reported a approximatively doubled VTE risk when compared to idiopathic controls (77).

Pregnancy can also trigger the most severe form of the syndrome, called catastrophic APS. This rare manifestation is characterized by multiorgan thrombosis, often in the microvasculature, occurring within a single week. Pregnancy is the precipitating factor in an estimated eight percent of cases, half of which occur during the pregnancy and half after (78, 79). Both maternal and perinatal mortality were high in one case-series, around fifty percent.

Interestingly, aPL do cross the placenta and newborns from APS mothers can test postive for these antibodies. Fortunately, this does not appear to cause thrombosis in the infants. Neurodevelopmental disorders have been observed but it is unclear whether there is an increased risk, let alone a causal relation (78, 80).

### Prevention and Treatment of Venous Thrombosis

The risk for pregnancy-related thrombosis necessitates prevention using anticoagulants. No trials have assessed different strategies for secondary thrombosis prophylaxis specifically in pregnant APS patients. But even under dual anticoagulant therapy with LDA and LMWH, pregnancy carries a high risk for thrombosis recurrence (81). Experts agree on treating all APS patients with previous thrombosis with therapeutic dose LMWH and LDA during pregnancy (75, 82). Women with obstetric APS are treated with a prophylactic dose during pregnancy and the puerperium. Vitamin K antagonists cross the placenta and can be teratogenic and cause fetal hemorrhage (83). They should therefore be replaced with LMWH as soon as pregnancy is confirmed. Based on data from animal studies, direct oral anticoagulants are also deemed unsafe during pregnancy and lactation (84). Moreover, data from clinical trials outside of pregnancy suggests these anticoagulants have inferior effectiveness compared to vitamin K antagonists, and at least for high-risk patients with arterial thrombosis they are not recommended (85, 86). Direct oral anticoagulants, if prescribed to APS patients, are ideally replaced by LMWH preconceptionally. This recommendation is largely based on the uncertainty about the teratogenicity of these agents (84), which may leave room for an alternative approach in patients with regular menses. If there is a strong preference to avoid long duration LWMH treatment (from the undefined preconceptional period until postpartum), frequent pregnancy testing in case of delayed menses and direct switching to LWMH upon a positive test may be preferred by a well-counseled patient.

New thrombosis occurring during pregnancy in an obstetric APS patient is also treated with LWMH. Catastrophic APS triggered by pregnancy is treated with a combination of intravenous heparin, glucocorticoids and either intravenous immunoglobulins or plasma exchange. Due to the nature of this manifestation, no trials are available, and treatment is based on expert opinion (87). Delivery should be considered, although it is not known whether this improves outcomes (88).

APS patients undergoing assisted reproductive technology procedures are also at high-risk for thrombosis (89). LMWH is recommended, at the same dose as what would be prescribed during a pregnancy in that individual patient (75, 82). The thrombotic risk is thought to be caused by the high estrogen levels. For this same reason, estrogen containing contraception is discouraged in women with APS (75, 82).

## Pregnancy Morbidity

### Epidemiology

The other clinical hallmark of APS aside from thrombosis, is obstetrical morbidity. A systematic review of the literature on APS antibody frequencies has shown that 6% of patients with APS related pregnancy morbidity are antibody positive (90). When restricting the analysis to studies that confirmed the diagnosis according to current criteria, the frequency ranged from 0 to 29%. The strength of the association seems to differ between the different obstetrical manifestations. In a European registry study of aPL-positive women, most of whom had APS according to the classification criteria, 54% had a history of recurrent miscarriage (91). However, the baseline risk of a single recognized pregnancy ending in miscarriage is already as high as 13% (92). Although recurrent miscarriage is a part of the classification criteria, the association with aPL is a matter of debate (93). Comparisons of observational studies on the topic are hampered by variation in the number and timing of pregnancy losses, aPL testing, and whether other causes for miscarriage were excluded. An extensive systematic review of these studies does suggest that the risk of early pregnancy losses is tripled in the presence of LAC and/or aCL (94). The same study reported risk increases with LAC for second trimester [OR 14.3 (95% CI 4.7–43.2)] and third trimester [OR 2.4 (95% CI 0.81–7.0)] pregnancy loss, and with aCL for third trimester loss [OR 3.3 (95% CI 1.6–6.7)]. A recent systematic review found odds ratios for late pregnancy loss ranging from 4.3 to 23, depending on the type of antibody (95). The third obstetric classifying manifestation of APS are placenta-mediated complications leading to premature birth, specifically pre-eclampsia, eclampsia and placental insufficiency. The frequency of pre-eclampsia in APS pregnancies is reported from 10 to 48% (96). Conversely, about 1 in 7 cases of pre-eclampsia may be APS-associated. The frequency of placental insufficiency is about 30%.

### Prevention of Pregnancy Morbidity

One question related to therapy for obstetric APS is whether a single treatment strategy is optimal for all the different manifestations. The 2020 American College of Rheumatology (ACR) Guidelines on the topic strongly recommends treating pregnant women with APS without prior thrombosis, with prophylactic heparin or LMWH, together with LDA (82). No distinction is made between prior APS manifestations. For patients with ≥ 2 prior early losses the evidence is summarized by a Cochrane Review (69). Meta-analysis of five trials produced a relative risk of live birth of 1.3 (95% CI 1.1–1.5) for heparin plus aspirin vs. aspirin alone. The certainty of evidence was judged low. Aspirin is started preconceptionally and heparin as soon as pregnancy is confirmed. LMWH are usually prescribed instead of unfractionated heparin because of convenience. A direct comparison in two small trials showed no difference (97, 98).

The European Alliance of Associations for Rheumatology does differentiate in its recommendations between women with late or recurrent early losses and women with preterm delivery due to placenta mediated complications (75). For the former, the recommendation parallels the recommendations made by the ACR. For the latter, it recommends either aspirin alone or in combination with prophylactic dose heparin. For this patient subgroup, one trial randomized between the two treatment strategies. It was unfortunately underpowered due to recruitment issues and did not show a difference in efficacy. There were no events in the LMWH plus aspirin group and two in the aspirin only group (99).

In analogy to systemic lupus erythematosus and based on a retrospective observational study, the ACR also recommends treating pregnant APS patients with hydroxychloroquine. This strategy is being evaluated in ongoing trials (100). Interestingly LMWH and LDA are also thought to act through non-antithrombotic (i.e., immunomodulatory) functions (101, 102), as hydroxychloroquine (103), but evidence is not conclusive to date (104). Another immunomodulatory therapeutic strategy under investigation is TNF-alpha inhibition by certolizumab pegol (NCT03152058).

## Discussion

Despite clear classification criteria, APS remains a complex disease, as highlighted by the large body of work implicating a wide range of cell types, signaling pathways and plasma proteases in its pathophysiology. A single key event within the pathophysiological pathway has however not yet been undisputedly pinpointed, although recent work does identify a new cell membrane lipid complex which links the antibody formation with induction of thrombosis and pregnancy morbidity.

Likewise, the exact origin of aPL remains an open question. Molecular mimicry has been suspected for a long time, but robust evidence linking the targeted autoantigens with intestinal microbe-expressed proteins were only recently reported and deserve further investigation.

Pregnancy is an important second hit in APS. It frequently provokes thrombosis, requiring secondary and sometimes primary thromboprophylaxis. A careful risk assessment is required. Similarly, in women in whom APS previously presented with pregnancy morbidity, secondary thromboprophylaxis is essential.

Trials have been performed to determine the optimal treatment strategy, but overall did not produce unequivocal results. Variations in patient populations, aPL testing and treatment are part of the explanation. Given the suboptimal efficacy and safety of anticoagulants and the non-coagulation-related mechanisms also involved in the pathophysiology, new non-anticoagulant based treatments are under investigation.

## Author Contributions

MK and TM wrote sections of the manuscript. Both authors contributed to manuscript revision, read, and approved the submitted version.

## Conflict of Interest

The authors declare that the research was conducted in the absence of any commercial or financial relationships that could be construed as a potential conflict of interest.

## Publisher's Note

All claims expressed in this article are solely those of the authors and do not necessarily represent those of their affiliated organizations, or those of the publisher, the editors and the reviewers. Any product that may be evaluated in this article, or claim that may be made by its manufacturer, is not guaranteed or endorsed by the publisher.
